# Effectiveness of multidisciplinary psychiatric home treatment for elderly patients with mental illness: a systematic review of empirical studies

**DOI:** 10.1186/s12888-019-2369-z

**Published:** 2019-12-03

**Authors:** Günter Klug, Manuela Gallunder, Gerhard Hermann, Monika Singer, Günter Schulter

**Affiliations:** 1 Society for Mental Health Promotion, Plüddemanngasse 45, A-8010 Graz, Austria; 2 Society for Mental Health Promotion, Hasnerplatz 4, A-8010 Graz, Austria; 3 Society for Mental Health Promotion, Plüddemanngasse 33, A-8010 Graz, Austria; 4 Department of Psychology, Biological Psychology Unit, University of Graz, Universitätsplatz 2, A-8010 Graz, Austria

**Keywords:** Mental illness, Multidisciplinary psychogeriatric home treatment, Elderly, Community mental health, Systematic review

## Abstract

**Background:**

The vast majority of older people with mental illness prefer to live independently in their own homes. Barriers caused by the health care system often prevent adequate, adapted treatments. With regard to the increasing ageing of the population, the determination of effective, age-appropriate service models for elderly patients with mental illness is clearly required. The aim of this review is to examine and to evaluate multidisciplinary psychogeriatric treatment models that include home visits, particularly with regard to the effects on psychiatric symptoms, social and mental health rehabilitation and quality of life.

**Methods:**

A systematic review was carried out of empirical studies with participants who were diagnosed with a mental illness according to ICD-10, aged 60 years or older, and who were living at home. The inclusion criteria comprised a duration of intervention of at least 12 weeks and a minimum of two interventions and domiciliary visits delivered by a multidisciplinary team. The online databases Medline, PsychInfo, Web of Science, Cochrane Register of Controlled Trials, and Google Scholar, as well as hand search, were used to search for relevant studies published between 1996 and 2016. An additional search was performed for studies published between 2016 and 2019. After removing duplicates, abstracts were screened and the remaining articles were included for full-text review.

**Results:**

Of the 3536 records discovered in total, 260 abstracts appeared to be potentially eligible. Of these, 30 full-text articles were assessed for eligibility. For the additional search 415 records and abstracts were screened and 11 articles were read full text. Finally, only three studies fully met the inclusion criteria for this review. The results indicate that psychogeriatric home treatment is associated with significant improvements of psychiatric symptoms and psychosocial problems, fewer admissions to hospital and nursing homes, as well as lower costs of care.

**Conclusions:**

Psychogeriatric home treatment has positive effects on older people with mental illness. However, these findings are based upon a small number of studies. The need for further research, especially to specify the effective factors in psychogeriatric home treatment, is clearly indicated.

## Background

Only a relatively small number of people aged 65 and over are long-term residents of a nursing or residential care home, for example 3.6% of the US 65+ population in 2011 [1]. The vast majority of older people with mental illness, about 80%, live independently in their own homes [2]. A considerable proportion of all non-institutionalised people aged 65+, about 28% of older persons in the United States [1] and 36% in Great Britain [3], live alone.

US 65+ population data show that 9.2% are considered to be homebound [4]. Psychiatric disorders are common in this group (40.5%) [5]. There are numerous mental disorders which affect the homebound elderly at a high rate [6]. This segment of population is severely disadvantaged and in many cases unable to access mental health treatment, including barriers posed by the health care system itself [7–10]. Cole and colleagues found back in 1996 that up to 90% of older people with depression receive no adequate treatment [11]. In 2015, the Royal College of Psychiatrists in the UK estimated that about 85% of older people with depression receive no help at all [3].

There is a worldwide lack of formal evaluation of psychiatric services for older people [12] and an increasing need to determine effective, age-appropriate service models for elderly patients with severe mental illnesses [7]. This is especially so because not treating or mistreating mental health problems exacerbates medical, functional and social problems, leading to higher rates of healthcare use, unneeded institutionalisation and increased mortality [13], and also a significant increase on health service costs [9].

Because of complex care needs, the most widely accepted model is a multidisciplinary, comprehensive, integrated service delivery to a defined catchment area [14]. However, a number of studies supporting these findings are primarily on persons settled in senior public housing or assisted living environments, and have not investigated persons in an independent living setting at home.

In addition, just as with healthy people, most elderly patients with mental health problems, especially dementia, live independently and prefer to continue living independently in their own familiar homes [15]. Care should be provided in patients’ homes as long as possible [16].

So, in this review we focused on treatment models and home visit programmes concerning the majority of older people with mental illness living in their own homes. The aim was to investigate whether multidisciplinary psychogeriatric home treatment for patients aged 60 years and over is more effective than usual care, by systematically reviewing empirical studies.

## Methods

As a quality assessment for reporting, the Preferred Reporting of Items for Systematic Reviews and Meta Analyses (PRISMA) statement was adopted to guide the conduct and reporting of the present systematic review [17]. A review protocol exists and can be made available by contacting the authors.

### Search strategy

We searched for studies examining the effects of multidisciplinary home treatment models on mentally ill adults who were aged 60 years or more, and who were living in their own homes.

Home treatment was defined as non-residential multidisciplinary psychiatric service that aimed to treat patients outside hospital or nursing homes to enable them to stay in their usual place of residence as long as possible [18]. The databases Medline, PsychInfo, Web of Science, and Cochrane Register of Controlled Trials were searched, starting in July 2012 up to August 2016, for relevant studies published between 1996 and 2016. We used the following search terms: mental health (e.g., mental illness, mental disease, geriatric psychiatry), age (e.g., old age, elderly people, older adults), type of treatment/setting (e.g., home treatment, home visiting programme, multidisciplinary team), outcome (e.g., effectiveness, health care costs, quality of care), type of study (e.g., randomized controlled trial, evaluation, evidence based), type of publication (original study, research article, review), and alternative search terms (health care needs, social needs). The Boolean search operators ‘AND’, ‘OR’, and ‘NOT’ were applied. Terms from the list of search terms (Additional file 2) were combined using ‘AND’ for the different categories (mental health; age; type of treatment/setting etc.) and ‘OR’ for synonyms and terms within the categories. For example, ‘mental illness AND home treatment AND multidisciplinary team AND effectiveness AND old age OR homebound’. The Boolean operator ‘NOT’ was used to specify the categories, for example, the age: ‘old age’ NOT ‘children’ NOT ‘youth’.

A full electronic search strategy for the Medline database is shown in Additional file 1, a full list of search terms in Additional file 2. An advanced search for additional articles was performed using Google scholar as well as hand search screening references listed in relevant studies. Results were not limited to studies published in English; studies published in German were also screened due to the authors’ language background. Keywords and inclusion criteria were defined by the research team in advance.

### Inclusion criteria

Studies with interventional study design (including RCTs, pre-post studies etc.) were eligible for inclusion if they provided the following criteria:

- Participants aged 60 years or older
- Minimum duration of intervention of 12 weeks to ensure the establishment of a sensible relationship based on trust with the patients
- Each participant has at least one psychiatric diagnosis according to ICD-10 [19] at the beginning of the study
- Intervention was implemented and delivered multidisciplinary, i.e. by more than one professional group (including psychiatrists, psychologists, psychotherapists, social workers, psychiatric nurses etc.)
- Mobile psychiatric care programme on the basis of domiciliary visits and psychiatric home treatment
- Participants were living at home alone or together with relatives
- Comparison of two or more intervention groups with regard to psychosocial or psychiatric symptoms.

Studies meeting the following criteria were not included:

- Mixed data without assessing the specific age group
- Studies on inpatients or participants who settled down in organized residential living systems, nursing homes, or receiving senior citizen housing or public housing (assisted living)
- Lack of psychiatric diagnosis and intervention
- Geriatric psychiatric assessment without integrated treatment (no input but screening or only surveying the needs and referring to therapies or treatments)
- Intervention by only one professional group
- No interventional study design
- Duplicate articles

### Data extraction and analysis

Data were extracted by three reviewers on the basis of a predefined data extraction form. This form was created to compare studies on different parameters in a standardised way, e.g., mean age etc.). The data extraction form - shown in Additional file 3 - was divided into the following sections:

- Introduction: data about unique identifier, author, title, journal, country, year and study setting (type, aim)
- Methods: data about study design, type of randomisation, time of examination, recruitment to study (inclusion and exclusion criteria), outcome measures, sample and research methodology
- Participants: data about age, gender, education level, religion, relationship status, income, living arrangements, medication, multimorbidity, patient history, ethnicity and diagnosis
- Intervention: data about treatment model, type of intervention, standardised programme, caseload, carer involvement, duration, intensity, setting, team profession, availability, description of usual service, time of examinations, referrals and costs
- Results: including limitations and strengths, methodology and statistics, and particular characteristics
- Study conclusions.

### Assessment of risk of bias

The risk of bias in all included studies was assessed by two review authors (GH and MS) using standard EPOC criteria [20]. We considered the following risk of bias domains: randomisation; allocation concealment; baseline data collection; incomplete outcome data; blinding; selective outcome reporting; contamination and other bias.

Overall, our electronic database search strategies identified 3526 records. Ten additional records were found by hand search, screening references listed in relevant studies. After broad screening of the titles, abstracts and keywords, and after removal of duplicates, 260 records appeared potentially eligible. They were screened by three researchers. Titles and abstracts were screened and the consensus of two reviewers was needed to exclude a study. The vast majority of these records did not meet the defined inclusion criteria concerning age and methodology. Thus, of the remaining abstracts, 30 articles were assessed as eligible for some aspect of the systematic review process. Two reviewers assessed each of the full reports, arriving at consensus regarding eligibility. Reviewers were GK, MG, and GH. Of these 30 articles, 27 articles were excluded because they could not be obtained (*n* = 1) or they did not meet the inclusion criteria for the following reasons: no assessment of psychiatric symptoms (*n* = 5), no multidisciplinary treatment (*n* = 4), participants were not living at home (n = 4) or because of a different target group (*n* = 13). In this way, only three studies met the inclusion criteria for this review completely.

### Additional search

In view of the lengthy writing up and publication process it was necessary to perform a search update. The database Medline was searched for the time period September 2016 to September 2019 and yielded 415 results. Titles and Abstracts were screened by two reviewers (GH and MS). Eleven studies seemed potentially interesting to fulfil our criteria and were read full text but had to be excluded because of the following reasons: no multidisciplinary treatment (*n* = 6), different target group (*n* = 3), and no assessment of psychiatric symptoms (*n* = 2). In the end we have found no further study that would fulfil our inclusion criteria entirely.

**Fig. 1:**
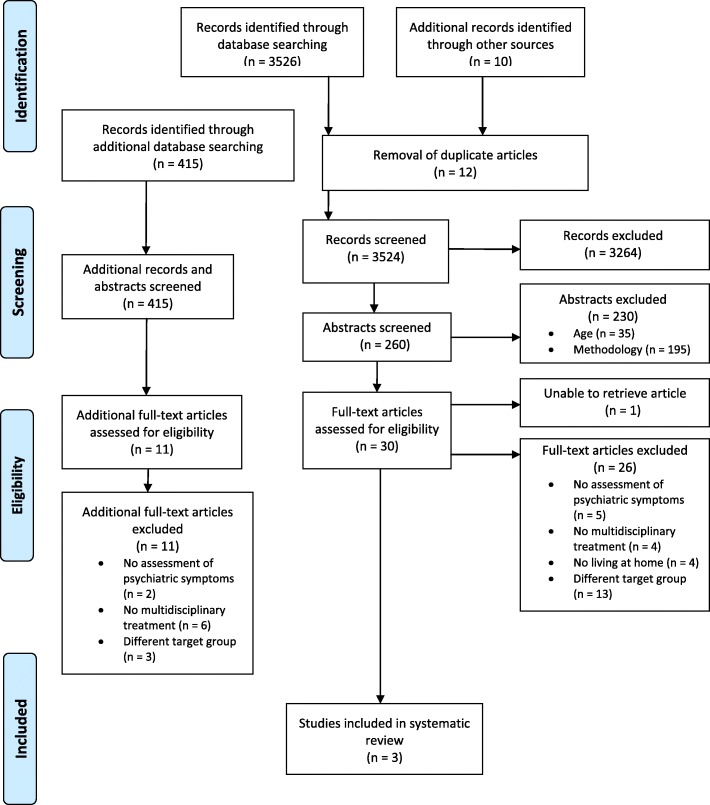
Flowchart of studies excluded and selected for systematic review

The corresponding flowchart is presented in Fig. 1.

## Results

### Overview of the included studies

The three studies which met the inclusion criteria were published in 1996, 2010 and 2014 respectively. One study was carried out in the Netherlands [23], one in Austria [22] and one in Great Britain [21]. All of them were randomized controlled trials. Participants in these studies were predominantly female and had a mean age between 74 and 81 years. The main diagnosis of the participants of two studies [21, 22] was depression, the main diagnoses of the participants of the third study were schizophrenia spectrum disorders, mood disorders and cognitive impairment [23]. All participants of the study intervention groups received psychiatric home care. None of the three studies focused on people with dementia considering our criteria.

### Characteristics of interventions

Table 1 shows the summary of the characteristics of interventions and outcomes.

**Table 1:** Summary of included studies

Studies	Banerjee et al. [21]	Klug et al. [22]	Stobbe et al. [23]
Sample	*n* = 69 age = 65 or over	*n* = 60 age = 65 or over	*n* = 62 age = 65 or over in the first year of research; afterwards minimum age = 60
Study type	RCT	RCT	RCT
Setting	UK /London As naturalistic as possible. Home	Austria /Graz Naturalistic service. Home	Netherlands/ Rotterdam In cooperation with an existing health care centre. TAU includes also multidisciplinary ACT teams. Home
Duration of intervention / Follow up	6 months (0/6) At baseline, and at 6 months after baseline	12 months (0/3/12) At baseline, at 3 months after baseline, and at 12 months after baseline	18 months (0/9/18) At baseline, at 9 months after baseline, and at 18 months after baseline
Intensity	No information	1–2 contacts per week In crisis situations up to 4 contacts per week.	According to the Guidelines for ACT Teams by SAaMHSA [50].
Intervention	Participants were not currently under psychiatric care. **Intervention group:** All were referred to a psychogeriatric team. The management plan for each subject on an individual basis was designed by the multidisciplinary team (see below) and implemented by the key worker who was always a doctor. He made no more visits than other team members. This could include any combination of physical interventions, e.g., prescription of antidepressants, physical review; psychological interventions, e.g., bereavement counselling or psychotherapy, family work; and social interventions, e.g., referral to a day centre, benefit check. **Control group:** General practitioner care. Letters were sent to general practitioners to say that their patient was participating in the study as a control but that this should not affect their management of him or her. The patients could be referred to as needed and they would be accepted by the team as normal if they were referred.	Access to all routine aspects of psychiatric care. **Intervention group:** In addition, all received geriatric home treatment over a 1-year-period. The individual care plan for each participant was designed by the multidisciplinary team (see below) working Mo-Fr 9-5 pm. Each Participant was visited once or twice a week. In crisis situations, up to four visits a week. Also phone contact with patients and carer. Components of geriatric home treatment were talks about self-esteem, coping resources and medication adherence; encouragement and practical support for the individual to establish and maintain social networks, increase social and leisure activities and cope with tasks of daily living; support of carers; and crisis intervention if required. **Control group:** Conventional psychiatric out-patient care. Individuals had free access to general practitioners and out-patient psychiatrists. They could also be referred to other services. Domiciliary visits were done rarely. In addition, all participants had an initial meeting with a psychologist for detailed information about all available health and social services and ways to access them.	**Intervention group:** ACTE (Assertive Community Treatment for the Elderly), a community-based treatment approach for outpatients whose SMI resulted in difficulties in daily living activities and social functioning often including problems with relationships, physical health, addiction, work, daytime activities and living conditions. ACT: Individualised services designed by the multidisciplinary team (see below) that provided psychiatric, somatic and rehabilitation treatment in the environment of the patient. Key features: assertive engagement, small and shared caseload (max. 1:10), based on treatment plan, community based and assertive services on a time unlimited basis with high contact frequency. **Control group:** TAU (Treatment as usual) was provided by three community mental health teams for elderly patients. Two of these teams were for patients with primary psychiatric disorders, one for patients with cognitive disorders. The teams provided regular mental health services including psychiatric care on an outreach basis. Various disciplines (community mental health nurses, a psychiatrist, and a psychologist) were individually responsible for the patients. High caseload (more than 25 patients per practitioner). All clinicians were specialised in treating elderly people.
Team Profession	Community psychiatric nurse, occupational therapist, senior and junior medical staff, social worker, psychologist	Psychiatrist and psychotherapist, psychologist, social worker, psychiatric nurse	Substance-abuse specialist, rehabilitation worker, social worker, psychiatric nurse, nurse specialized in somatic care, community mental health nurse, psychiatrist
Diagnosis	Major Depression in context with geriatric mental state –AGECAT system	Major Depression in context with GDS-15 score, GAF 21–60, MMSE > 27 [and living independently]	Severe mental illnesses including schizophrenia spectrum disorders, mood disorders, other disorders plus problems in daily functioning and engaging in treatment in context with Health of the Nation Outcome Scales for elderly people and exclusion of severe cognitive impairment.
Outcome measures	Selfcare(d) questionnaire. Multidimensional functional assessment. AGECAT (automatic geriatric examination for computer assisted taxonomy) Montgomery Asberg depression rating scale. Blinded follow up	Symptom levels of depression (GDS-15 Geriatric Depression Scale). Level of functioning (GAF Global Assessment of Functioning Scale). Level of subjective quality of life (SQOL based on BELP-KF Berlin Quality of Life Profile). Admissions to nursing homes and days spent there. Days spent in psychiatric inpatient care. Costs of care included intervention, days in nursing homes, days in psychiatric inpatient care. Not blinded. Self and observer rated	Primary outcome measures: Number of patients engaged in the first 3 months. Number of dropouts. Psychosocial functioning. Secondary outcome measures: Number of unmet needs. Number and Duration of inpatient psychiatric admission. Number of crisis contacts. Based on: HoNOS65+ (Health of the Nation Outcome Scales for elderly people). CANE, staff member version (Camberwell Assessment of Need for the Elderly). Measuring the model fidelity of ACT; Measuring the model fidelity of ACTE and TAU 2 years after start with DACTS (Dutch version of the Dartmouth Assertive Community Treatment Scale). Not blinded
Outcomes	Psychiatric team treatment at home was substantially more effective than general practitioner care alone in treating depression in this disabled elderly population. Significant beneficial follow up effects.	Patients receiving geriatric home treatment had significantly fewer symptoms of depression, better global functioning and a higher SQOL at 3 months and 12 months. Over 1 year they had significantly fewer admissions to nursing homes, spent less time in psychiatric in-patient care, and care costs were significantly lower.	ACTE had better results than TAU with regard to engaging patients into treatment within 3 months and fewer dropouts from treatment. Improvements but no significant differences in the other primary and secondary outcome variables, which means that ACTE did not produce better outcomes with respect to psychosocial functioning, unmet needs or mental health care use. Patients allocated to ACTE had significantly more often contact with mental healthcare workers. Hospital days and crisis contacts: These variables were not analysed statistically. Very few patients had been admitted or had had crisis contacts. Model fidelity: TAU teams had lower model fidelity scores, but model fidelity in ACTE was also only moderate. ACTE scored high on the small and shared caseload and time-unlimited services; maximum scores for community based services and assertive outreach. Low model fidelity concerning a vocational specialist and consumer provider in the team, the frequency of contact, the intensity of service, the intake rate, dual-disorders treatment groups,work with the support system, and responsibility for crisis services.

In all three studies the intervention was implemented by more than one professional group. The multidisciplinary teams (including psychiatrists, psychologists, psychotherapists, social workers, and psychiatric nurses) delivered treatment locally at home. Two studies evaluated the effectiveness of a psychogeriatric team intervention in the treatment of older people with depression living at home [21, 22]. The intervention programme in the study by Klug et al. [22] consisted of talks about self-esteem, coping resources and medication adherence, encouragement and practical support for the individual to establish and maintain social networks, increase social and leisure activities and cope with tasks of daily living, support of carers and crisis intervention. An individual care plan for each participant was developed [22]. Also in the study by Banerjee et al. [21] an individual management plan for each subject was formulated. Interventions included prescription of antidepressants, physical review, social measures, counselling or psychotherapy, family work, outreach referral, activities of daily living and living assessment [21]. The third study investigated whether an assertive community treatment for elderly patients (ACTE) with severe mental illness resulting in difficulties in daily living activities and social functioning, physical health, addiction, work, daytime activities and living conditions, is more successful than treatment as usual (TAU) in engaging patients into care within three months, preventing dropout from treatment, and producing better outcomes with respect to psychosocial functioning, unmet needs or mental health care use. Treatment as usual was provided by three community mental health teams which offered regular mental health services, including psychiatric care on an outreach basis. Intervention (ACTE) was characterised by a team approach, shared and smaller caseload, higher frequency of contact, and the direct provision of care in the form of individualised services in comparison to TAU [23]. The duration of interventions varied within the included studies from 6 to 12 months [21, 22] up to 18 months [23]. The intensity of the visits varied from 1 to a mean of 3 contacts a week [22, 23]. Klug et al. [22] arranged up to four contacts a week in crisis situations. The caseload was declared in only one study with a maximum of 10 patients per clinician [23]. To measure the effect size of the intervention, each study compared the results with the results of the control group. In all control groups the participants received usual services which differed slightly in the reviewed studies. The outcomes of the three studies were assessed with completely different instruments. Table 1 gives a detailed summary of the three studies.

### Characteristics of instruments

Depression was self-rated by Klug et al. [22] on the 15-item Geriatric depression scale [24]. Banerjee et al. [21] used the self-rating Selfcare(d) questionnaire [25] and the Montgomery Asberg depression rating scale [26]. Banerjee et al. [21] assessed the mental state using the geriatric mental state/AGECAT (automatic geriatric examination for computer assisted taxonomy) system [27, 28]. Klug et al. [22] also applied in advance (for preselection concerning exclusion criteria) the Mini-Mental-State Examination [29]. Further instruments were the Global Assessment of Functioning Scale [30], and the short form of the Berlin Quality of Life Profile (BELP-KF) [31] for assessing the subjective quality of life (SQOL). Stobbe et al. [23] used the Dutch version of the Health of the Nation Outcome Scales for elderly people (HoNOS65+) to assess the severity of psychosocial problems [32, 33]. To measure care needs, the short Dutch version of the Camberwell Assessment of Needs for the Elderly (CANE, stuff member version) was applied [34, 35]. The model fidelity was measured using the Dutch version of the Dartmouth Assertive Community Treatment Scale (DACTS) [36].

### Study outcomes

Two studies [21, 22] indicate that psychogeriatric home treatment reduces depressive symptoms. A significant difference and a positive impact of the intervention concerning global functioning, quality of life and care costs was also found in the study by Klug et al. [22].

Data regarding the medical necessity of an inpatient admission to hospital or nursing homes were only assessed by Klug et al. [22], and showed significantly lower scores in the intervention group. Stobbe et al. [23] identified an improvement in psychosocial functioning and a significant decrease in the total number of unmet needs in both groups, but no significant preference for the study group. Patients allocated to ACTE had significantly more often contact with mental healthcare and had fewer dropouts than those allocated to treatment as usual (Table 1). The authors give various reasons to explain the lack of differences regarding outcome in psychosocial functioning: a selection bias in TAU due to the differences in the number of patients; a selection bias in ACTE by preventing the dropout of patients who had worse prognoses than the others; TAU used components of ACTE; and the fact that ACTE did not include a psychologist in the team which may have limited its effectiveness. Results are presented in detail in Table 1.

### Risk of bias in included studies

The risk of bias for individual studies is reported in Table 2. Overall the studies were of reasonable quality with low risk of bias. However, concerning blinding there was a high risk of bias in two studies [22, 23] as Interviews or Ratings were not assessed blindly. Furthermore, Stobbe et al. [23] mention a selection bias as possible limitation. A potential attrition bias is discussed in the study by Banerjee et al. [21] and Stobbe et al. [23].

**Table 2:** Risk of bias

Risk of bias	Authors’ judgement; Banerjee et al. [21]	Authors’ judgement; Klug et al. [22]	Authors’ judgement; Stobbe et al. [23]
Random sequence generation (selection bias)	Low risk (Computer generated scheme)	Low risk (Random number table)	Low risk (Computer generated scheme)
Allocation concealment (selection bias)	Low risk (Computer system)	Low risk (Centralised randomisation scheme)	High risk (Authors discuss a selection bias as limitation)
Baseline outcome measurements similar	Low risk (Measured prior to the intervention)	Low risk (Measured prior to the intervention)	Low risk (Measured prior to the intervention)
Baseline characteristics similar	Low risk (No significant differences)	Low risk (No significant differences)	Low risk (No significant differences)
Incomplete outcome data (attrition bias)	High risk (The possibility of non-response bias as stated by the authors)	Low risk (Missing data was unlikely to overturn the study result)	High risk (High number of patients lost to follow-up)
Knowledge of the allocated interventions adequately prevented during the study	Low risk (Primary outcome variable assessed blindly)	High risk (Interviews were not assessed blindly)	High risk (Raters were not blind for the treatment condition)
Selective outcome reporting (reporting bias)	Low risk (All outcomes reported)	Low risk (All outcomes reported)	Low risk (All outcomes reported)
Protection against contamination	Low risk (Control group had no access to patient oriented intervention)	Low risk (Control group had no access to patient oriented intervention)	Low risk (Control group had no access to patient oriented intervention)
Other risks of bias	Low risk	Low risk	Low risk

## Discussion

This review gives an insight into the state of research in the field of outreach geriatric psychiatry in a purely domestic environment. As far as we know, this was one of the first reviews that specifically examined the research status for psychogeriatric home treatment directly in a home environment. Abendstern and colleagues [37] have also undertaken a review of this nature but they did not focus on interventions of 12 weeks or more. In contrast, previous reviews included community-based lower-threshold settings like senior centres or senior housing [9] or focused on psychotherapeutic interventions [38]. As already noted by Bruce et al. [9], there are only few RCTs, regardless of the fact, that the vast majority of older people with mental illness live at home.

The present review provides evidence regarding successful treatment strategies for older patients living at home. Data show significant positive effects on relevant parameters such as fewer symptoms of depression [21, 22], an improvement in global and psychosocial functioning [22, 23] and better quality of life [22]. Despite different survey instruments, about five years of difference in the average age and differences in the allocation, the findings by Banerjee et al. [21] with regard to improvement in depressive symptoms can be considered as confirmed by Klug et al. [22].

Stobbe et al. [23] could demonstrate positive effects of engaging with people with SMI. Furthermore, the findings discussed by Klug et al. indicate fewer admissions to nursing homes, fewer inpatient days spent in psychiatric hospitals as well as lower costs of care [22]. Thus, multidisciplinary psychiatric home treatment may also result in better economic efficiency than treatment as usual and so an implementation of this approach as part of standard care is certainly indicated.

### Strengths and limitations of the included studies

Strengths: All studies were pragmatic trials in routine services or were implemented in as natural a way as possible based on complex interventions. Banerjee et al. [21] clearly defined and assessed the main diagnoses, and the study was blinded. Klug et al. [22] used a mixture of self-rating and observer rating tools and included research on costs. Stobbe et al. [23] compared intervention measures with a comparatively high quality treatment as usual (TAU). Power calculation on sample size was done by Banerjee et al. [21] and Klug et al. [22]. Overall, all three studies showed considerable (though not all of them statistically significant) effects of improvement, despite the fact that there was only a small difference in treatment between ACTE and TAU in the study by Stobbe et al. [23] in the first place.

### Discussion in view of the literature

The lack of high-level studies to investigate interventions in a home environment is evident, especially with reference to dementia disorders. We found no studies fulfilling the inclusion criteria with focus on people living at home with dementia. The only longitudinal study by Carbone et al. [39] which was based on multidisciplinary psychiatric home treatment showed encouraging results at three months follow up but could not be included due to the lack of a control group. However, Challis and colleagues [40] evaluated a model of intensive case management for people with dementia based in a community-based mental health service for older people and found previous findings confirmed that the most effective case management interventions are those targeted on a highly specific client group.

Dementia related studies currently focus on caregivers, for example that by Van Knippenberg et al. [41]. A recently published systematic review demonstrated a lack of consistency in relation to the dementia ascertainment methodology [42].

The complexity and time demands of conducting randomized trials in this setting may explain the comparatively large number of studies reporting qualitative and observational outcome data [9].

It should also be considered why older patients with severe mental disorders are difficult to reach and engage [23]. Perhaps the treatment is often not really ‘low-threshold’ or is based on inappropriate contents. To our knowledge, treatments based on trust and strong confidence, burden oriented time resources and continuity between caretaker and patient achieve best results [22]. The dropout rate [23] could be reduced by an expanded psychiatric home treatment. The rate is still high, compared to the other two reviewed studies. This could be due to the fact that the contact was made, at least in part, in the first three months. The model fidelity was weak in the number of contacts, which could be a further reason.

All authors mentioned the small number of participants, but only Stobbe et al. [23] had problems in reaching the predefined power due to dropouts. In that study, the recruiting problems, the high dropout rate and the moderate model fidelity in ACTE weakened the power to detect changes. The high level of TAU (which had a few elements in common with ACT) may explain why there were considerable effects but no significant differentiation between the groups [23].

Overall, more studies of that kind are needed in several aspects to prove the results. For example, costs were only assessed by Klug et al. [22]. In general, there should not only be a focus on mental but on all health care costs, as because of multimorbidity, physical and psychiatric symptoms are mutually dependent and have therefore to be perceived in all their complexity.

The measurement tool for psychosocial functioning has to be discussed. Stobbe et al. pointed out that the sum score of the instrument used has been criticized for not properly measuring change in psychosocial functioning, ratings were not blinded, and not every assessment was filled out after the face-to-face contact with the patient [23].

Although complex interventions were performed in all three studies, a basic description of the contents of the interventions was only presented by Klug et al. [22] and Stobbe et al. [23]. Only Klug et al. [22] assessed data about the concrete application of the intervention contents in detail.

However, regarding treatment in relation to the control group, no detailed specification was given in the study by Banerjee et al. and Klug et al. In these two studies [21, 22], the control group received treatment as usual, but there was no specific information about the treatment the participants actually used or whether they used any treatment at all. Intervention and treatment as usual have to be defined in a more specific way for comparability. It is not easy to come to clear conclusions due to the heterogeneity of the studies regarding diagnoses, survey instruments and target differences in the primary outcomes. So, conclusions are only partially derivable.

The sample characteristics also differed as follows: One study considered those who had already received homecare but no psychiatric care [21], the second study also accepted participants without homecare or in outpatient psychiatric treatment [22]. The third study focused on people with SMI to connect them with psychiatric home treatment [23]. In two studies [21, 22], the control group did not receive any psychiatric home treatment at all. In the third study, a specially designed geriatric psychiatric home treatment based on a lower caseload (≤10) was compared with an assertive community treatment (caseload > 25) [23].

The caseload is not known in two studies [21, 22]. Little is known about the characteristics of the study participants in general. The three studies are comparable concerning the fact that two-thirds to more than three-quarters of the people were living alone. The proportion is highest in the study by Stobbe et al. [23] with 84.4% in the intervention group and 90% in the treatment as usual group.

With an average age of about 81 years (80.4 years in intervention group and 81 years in control group), participants in the study by Banerjee et al. [21] were obviously older than participants in both of the other two studies with an average age of about 75 years (74.4 years in ACTE and 75.1 years in TAU) in the study by Stobbe et al. [23] and (74.3 years in intervention group and 75.5 years in control group) in the study by Klug et al. [22]).

### Strengths and limitations of this review

The strength of the present review is the focus on one specific topic with exact predefined inclusion criteria to ensure the comparability of the data as far as possible. The inclusion criteria were very strict in order to maximise the comparability and to focus on the target group very accurately to get a clear picture. So, only three studies met the defined criteria. Nevertheless, we did not achieve the desired comparability. Therefore, this strength is at the same time a limitation as well, as conclusions based on the comparison of these studies are limited because of the differences in diagnosis, used instruments, control groups and vague study descriptions. This shows that the comparability definitely needs to be improved.

A further potential limitation of this review is the extraordinarily long period of investigation. The start of the review was in 2012; an additional literature search had to be performed between 2014 and 2016 due to limited resources which subsequently led to a big time delay.

### Considerations for future studies

The intensity of home visits varies in diverse studies from four visits a year [43] to four contacts a week [22]. The number of visits for an adequate and effective supply has to be determined in the context of the targets of treatment. If it is, for example, a primary objective to ensure outreach living in the case of severe mental illness while minimising inpatient treatment; a high contact rate and adequate resources have to be provided, as practised by Klug et al. [22].

Treatment in primary health care for older people requires a multi-professional team approach. Because there is no standardised definition of a multi-professional team, comparability is difficult. The physician is often the project manager, but the leader should be selected not only because of his or her professional background but also because of his or her interests, social and emotional competences, and personality [44].

In contrast to multi-professional treatments, several studies have been found based on mono-professional treatments with a multi-professional background [45, 46]. They are also worth discussion.

## Conclusions

More than 20 years ago, Wertheimer [16] noted that in most countries community service models for older people are less developed than those for individuals in middle, respectively working age. Therefore, only a few studies existed. Nowadays, long-term studies and studies on specific diagnose groups are still missing. The study by Tucker et al. [47] suggested that if enhanced community services were available, a significant minority of inpatients could be more appropriately supported in their own homes at a cost considerably lower than that currently incurred. Sorrell [48] underlined the importance of health care professionals, researchers, and policy makers, to continue to advocate for a mental health care system that is accessible and effective for older adults in the community, and summed up: ‘We can do better’ (p.1).

Replications of existing studies are clearly required. Larger sample sizes and longer follow up periods are needed as well as better descriptions to enable identification of the most relevant factors of geriatric home treatment.

Although studies in this topic are struggling with the complexity of the target group, more research needs to be carried out, due to the importance concerning demography, quality of life for the patients, and economic relevance, especially on different psychiatric diagnoses. To get good and comparable results some factors such as, e.g., multi-professional teams, treatment as usual, and intervention should be standardised and the instruments adapted to the specifics for older people. Community-dwelling and homebound elderly should be differentiated [9 p. 1056]. So far multi-professional home treatment has focused mainly on younger adults [49].

Overall, we conclude that investment in an adequate multidisciplinary psychiatric home treatment may lead to better clinical and social outcomes, combined with greater cost efficiencies.

## Supplementary information

**Additional file 1.** Medline search strategy.

**Additional file 2.** List of search terms.

**Additional file 3.** Data extraction form.

## Data Availability

Data sharing is not applicable to this article as no datasets were generated or analysed.

## Abbreviations

ACT: Assertive Community Treatment
ACTE: Assertive Community Treatment for Elderly
AGECAT: Automated Geriatric Examination for Computer Assisted Taxonomy
AoA: Administration of Aging
BELP-KF: Berlin Quality of Life Profile-Kurzform (short form)
CANE: Camberwell Assessment of Need for the Elderly
CAU: Care as usual
DACTS: Dartmouth Assertive Community Treatment Scale
EPOC: Effective Practice and Organisation of Care
GAF: Global Assessment of Functioning
GDS: Geriatric Depression Scale
HoNOS65+: Health of the Nation Outcome Scales for elderly people
MMSE: Mini Mental State Examination
PRISMA: Preferred Reporting Items for Systematic Reviews and Meta-Analyses
RCT: Randomised Controlled Trial
SAaMHSA: Substance Abuse and Mental Health Services Administration
SQOL: Subjective Quality Of Life
TAU: Treatment as usual
UK: United Kingdom
US: United States
