# Supplementary material for: Effectiveness of multidisciplinary psychiatric home treatment for elderly patients with mental illness: a systematic review of empirical studies
Source: BMC Psychiatry. 2019 Dec 3;19:382. doi: 10.1186/s12888-019-2369-z (PMC6889722; doi:10.1186/s12888-019-2369-z)
Supplement: Supplementary file 1 — Additional file 1. Medline search strategy. [file 12888_2019_2369_MOESM1_ESM.doc]

**Suchformulierung:** (((FT=geriatric mental health AND FT=home treatment ) OR FT=home based support ) OR FT=outpatient ) AND FT=multidisciplinary team

**Trefferzahlen in den einzelnen Datenbanken [
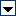
](https://portal.dimdi.de/websearch/servlet/FlowController/Search)**

| **Kürzel** | **Name** | **Info** | **Trefferzahl** |
| --- | --- | --- | --- |
| ME60 | MEDLINE | [Info](http://www.dimdi.de/de/db/dbinfo/me66.htm) | 326 |

Formularbeginn

**Gefundene Dokumente: 326**

Formularende

**Suchformulierung:** ((((FT=geriatric mental health AND FT=home treatment ) OR FT=home based support ) OR FT=outpatient ) AND FT=multidisciplinary team ) AND PY=2014 to 2016 AND (ct=aged OR ct="aged, 80 and over")

**Trefferzahlen in den einzelnen Datenbanken [
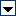
](https://portal.dimdi.de/websearch/servlet/FlowController/Search)**

| **Kürzel** | **Name** | **Info** | **Trefferzahl** |
| --- | --- | --- | --- |
| ME60 | MEDLINE | [Info](http://www.dimdi.de/de/db/dbinfo/me66.htm) | 17 |

Formularbeginn

**Gefundene Dokumente: 17**

Formularende

**Suchformulierung:** ((((FT=effect AND AU="multidisciplinary team") AND FT=community based ) OR FT=home treatment ) AND FT=old age psychiatry ) AND PY=2014 to 2016 AND (ct=aged OR ct="aged, 80 and over")

**Trefferzahlen in den einzelnen Datenbanken [
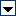
](https://portal.dimdi.de/websearch/servlet/FlowController/Search)**

| **Kürzel** | **Name** | **Info** | **Trefferzahl** |
| --- | --- | --- | --- |
| ME60 | MEDLINE | [Info](http://www.dimdi.de/de/db/dbinfo/me66.htm) | 1 |

Formularbeginn

**Gefundene Dokumente: 1**

Formularende

**Suchformulierung:** ((((FT=effect AND AU="multidisciplinary team") AND FT=community based ) OR FT=home treatment ) AND FT=old age psychiatry ) AND (ct=aged OR ct="aged, 80 and over")

**Trefferzahlen in den einzelnen Datenbanken [
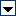
](https://portal.dimdi.de/websearch/servlet/FlowController/TabTitles?uid=4)**

| **Kürzel** | **Name** | **Info** | **Trefferzahl** |
| --- | --- | --- | --- |
| ME60 | MEDLINE | [Info](http://www.dimdi.de/de/db/dbinfo/me66.htm) | 2 |

Formularbeginn

**Gefundene Dokumente: 2**

Formularende

**Suchformulierung:** ((((FT=geriatric AND AU="mental health") AND FT=home visiting program ) OR FT=home visit ) AND FT=multidisciplinary ) AND PY=2016 AND (ct=aged OR ct="aged, 80 and over")

Es wurden keine mit Ihrer Suchanfrage übereinstimmenden Dokumente gefunden.

**Suchformulierung:** ((((FT=geriatric AND AU="mental health") AND FT=home visiting program ) OR FT=home visit ) AND FT=multidisciplinary ) AND (ct=aged OR ct="aged, 80 and over")

**Trefferzahlen in den einzelnen Datenbanken [
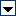
](https://portal.dimdi.de/websearch/servlet/FlowController/Search)**

| **Kürzel** | **Name** | **Info** | **Trefferzahl** |
| --- | --- | --- | --- |
| ME60 | MEDLINE | [Info](http://www.dimdi.de/de/db/dbinfo/me66.htm) | 13 |

Formularbeginn

**Gefundene Dokumente: 13**

Formularende

**Suchformulierung:** (((FT=effectiveness AND AU="home treatment") AND FT=old age psychiatry ) OR FT=assertive community treatment ) AND FT=multidisciplinary team

**Trefferzahlen in den einzelnen Datenbanken [
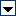
](https://portal.dimdi.de/websearch/servlet/FlowController/Search)**

| **Kürzel** | **Name** | **Info** | **Trefferzahl** |
| --- | --- | --- | --- |
| ME60 | MEDLINE | [Info](http://www.dimdi.de/de/db/dbinfo/me66.htm) | 12 |

Formularbeginn

**Gefundene Dokumente: 12**

Formularende

**Suchhistorie [
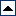
](https://portal.dimdi.de/websearch/servlet/FlowController/TabSearch?uid=26)**

Formularbeginn

| **Nr.** | **Suchformulierung** | **Trefferzahl** | **Aktionen** |
| --- | --- | --- | --- |
| 10 | 3 AND 8 AND 9 | 0 | [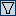](https://portal.dimdi.de/websearch/servlet/FlowController/DisplayLimits?uid=27&id=10)[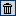](https://portal.dimdi.de/websearch/servlet/FlowController/DeleteResult?uid=27&id=10) |
| 9 | 2 AND 8 | 0 | [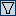](https://portal.dimdi.de/websearch/servlet/FlowController/DisplayLimits?uid=27&id=9)[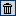](https://portal.dimdi.de/websearch/servlet/FlowController/DeleteResult?uid=27&id=9) |
| 8 | [((((FT=effectiveness AND AU="home treatment") AND FT=old age psychiatry ) OR FT=assertive community treatment ) AND FT=multidisciplinary team ) AND PY=2014 to 2016](https://portal.dimdi.de/websearch/servlet/FlowController/SelectResult?uid=27&id=8) | 4 | [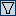](https://portal.dimdi.de/websearch/servlet/FlowController/DisplayLimits?uid=27&id=8)[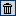](https://portal.dimdi.de/websearch/servlet/FlowController/DeleteResult?uid=27&id=8) |
| 7 | [(((FT=effectiveness AND AU="home treatment") AND FT=old age psychiatry ) OR FT=assertive community treatment ) AND FT=multidisciplinary team](https://portal.dimdi.de/websearch/servlet/FlowController/SelectResult?uid=27&id=7) | 12 | [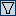](https://portal.dimdi.de/websearch/servlet/FlowController/DisplayLimits?uid=27&id=7)[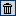](https://portal.dimdi.de/websearch/servlet/FlowController/DeleteResult?uid=27&id=7) |
| 6 | (((FT=effectiveness AND AU="home treatment") AND FT=old age psychiatry ) AND FT=assertive community treatment ) AND FT=multidisciplinary team | 0 | [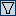](https://portal.dimdi.de/websearch/servlet/FlowController/DisplayLimits?uid=27&id=6)[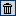](https://portal.dimdi.de/websearch/servlet/FlowController/DeleteResult?uid=27&id=6) |
| 5 | [((((FT=geriatric AND AU="mental health") AND FT=home visiting program ) OR FT=home visit ) AND FT=multidisciplinary ) AND (ct=aged OR ct="aged, 80 and over")](https://portal.dimdi.de/websearch/servlet/FlowController/SelectResult?uid=27&id=5) | 13 | [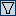](https://portal.dimdi.de/websearch/servlet/FlowController/DisplayLimits?uid=27&id=5)[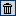](https://portal.dimdi.de/websearch/servlet/FlowController/DeleteResult?uid=27&id=5) |
| 4 | ((((FT=geriatric AND AU="mental health") AND FT=home visiting program ) OR FT=home visit ) AND FT=multidisciplinary ) AND PY=2016 AND (ct=aged OR ct="aged, 80 and over") | 0 | [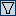](https://portal.dimdi.de/websearch/servlet/FlowController/DisplayLimits?uid=27&id=4)[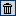](https://portal.dimdi.de/websearch/servlet/FlowController/DeleteResult?uid=27&id=4) |
| 3 | [((((FT=effect AND AU="multidisciplinary team") AND FT=community based ) OR FT=home treatment ) AND FT=old age psychiatry ) AND (ct=aged OR ct="aged, 80 and over")](https://portal.dimdi.de/websearch/servlet/FlowController/SelectResult?uid=27&id=3) | 2 | [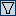](https://portal.dimdi.de/websearch/servlet/FlowController/DisplayLimits?uid=27&id=3)[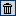](https://portal.dimdi.de/websearch/servlet/FlowController/DeleteResult?uid=27&id=3) |
| 2 | [((((FT=effect AND AU="multidisciplinary team") AND FT=community based ) OR FT=home treatment ) AND FT=old age psychiatry ) AND PY=2014 to 2016 AND (ct=aged OR ct="aged, 80 and over")](https://portal.dimdi.de/websearch/servlet/FlowController/SelectResult?uid=27&id=2) | 1 | [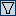](https://portal.dimdi.de/websearch/servlet/FlowController/DisplayLimits?uid=27&id=2)[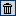](https://portal.dimdi.de/websearch/servlet/FlowController/DeleteResult?uid=27&id=2) |
| 1 | Datenbankauswahl: |  |  |

Formularende

**Suchhistorie [
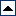
](https://portal.dimdi.de/websearch/servlet/FlowController/TabSearch?uid=33)**

Formularbeginn

| **Nr.** | **Suchformulierung** | **Trefferzahl** | **Aktionen** |
| --- | --- | --- | --- |
| 13 | [((((FT=evidence AND AU="home treatment") AND FT=old age psychiatry ) OR FT=outpatient treatment ) AND FT=multidisciplinary team ) AND (ct=aged OR ct="aged, 80 and over")](https://portal.dimdi.de/websearch/servlet/FlowController/SelectResult?uid=34&id=13) | 1 | [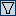](https://portal.dimdi.de/websearch/servlet/FlowController/DisplayLimits?uid=34&id=13)[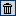](https://portal.dimdi.de/websearch/servlet/FlowController/DeleteResult?uid=34&id=13) |
| 12 | [(((FT=evidence AND AU="home treatment") AND FT=old age psychiatry ) OR FT=outpatient treatment ) AND FT=multidisciplinary team](https://portal.dimdi.de/websearch/servlet/FlowController/SelectResult?uid=34&id=12) | 13 | [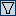](https://portal.dimdi.de/websearch/servlet/FlowController/DisplayLimits?uid=34&id=12)[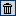](https://portal.dimdi.de/websearch/servlet/FlowController/DeleteResult?uid=34&id=12) |
| 11 | [((((FT=evaluation AND AU="home treatment") AND FT=old age psychiatry ) OR FT=outpatient treatment ) AND FT=multidisciplinary team ) AND (ct=aged OR ct="aged, 80 and over")](https://portal.dimdi.de/websearch/servlet/FlowController/SelectResult?uid=34&id=11) | 1 |  |

Formularende

**Suchhistorie [
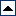
](https://portal.dimdi.de/websearch/servlet/FlowController/TabSearch?uid=40)**

Formularbeginn

| **Nr.** | **Suchformulierung** | **Trefferzahl** | **Aktionen** |
| --- | --- | --- | --- |
| 14 | [(((FT=quality of care AND AU="home treatment") AND FT=old age psychiatry ) OR FT=outpatient treatment ) AND FT=multidisciplinary team](https://portal.dimdi.de/websearch/servlet/FlowController/SelectResult?uid=41&id=14) | 13 | [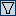](https://portal.dimdi.de/websearch/servlet/FlowController/DisplayLimits?uid=41&id=14) |

Formularende

**Suchhistorie [
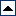
](https://portal.dimdi.de/websearch/servlet/FlowController/TabSearch?uid=54)**

Formularbeginn

| **Nr.** | **Suchformulierung** | **Trefferzahl** | **Aktionen** |
| --- | --- | --- | --- |
| 20 | [((((FT=evidence AND AU="home based support") AND FT=elderly ) OR FT=homebound ) AND FT=multidisciplinary ) AND PY=2014 to 2016 AND (ct=aged OR ct="aged, 80 and over")](https://portal.dimdi.de/websearch/servlet/FlowController/SelectResult?uid=55&id=20) | 1 | [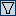](https://portal.dimdi.de/websearch/servlet/FlowController/DisplayLimits?uid=55&id=20)[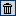](https://portal.dimdi.de/websearch/servlet/FlowController/DeleteResult?uid=55&id=20) |
| 19 | [((((FT=evidence AND AU="home based support") AND FT=elderly ) OR FT=homebound ) AND FT=multidisciplinary ) AND (ct=aged OR ct="aged, 80 and over")](https://portal.dimdi.de/websearch/servlet/FlowController/SelectResult?uid=55&id=19) | 20 | [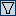](https://portal.dimdi.de/websearch/servlet/FlowController/DisplayLimits?uid=55&id=19)[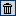](https://portal.dimdi.de/websearch/servlet/FlowController/DeleteResult?uid=55&id=19) |
| 18 | ((((FT=evidence AND AU="home based support") AND FT=old age psychiatry ) OR FT=homebound ) AND FT=psychogeriatric team ) AND (ct=aged OR ct="aged, 80 and over") | 0 | [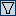](https://portal.dimdi.de/websearch/servlet/FlowController/DisplayLimits?uid=55&id=18)[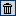](https://portal.dimdi.de/websearch/servlet/FlowController/DeleteResult?uid=55&id=18) |
| 17 | (((FT=effectiveness AND AU="home treatment") AND FT=old age psychiatry ) OR FT=homebound ) AND FT=psychogeriatric team | 0 | [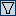](https://portal.dimdi.de/websearch/servlet/FlowController/DisplayLimits?uid=55&id=17)[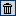](https://portal.dimdi.de/websearch/servlet/FlowController/DeleteResult?uid=55&id=17) |
| 16 | ((((FT=effects AND AU="home treatment") AND FT=old age psychiatry ) OR FT=homebound ) AND FT=psychogeriatric team ) AND (ct=aged OR ct="aged, 80 and over") | 0 | [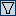](https://portal.dimdi.de/websearch/servlet/FlowController/DisplayLimits?uid=55&id=16)[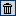](https://portal.dimdi.de/websearch/servlet/FlowController/DeleteResult?uid=55&id=16) |
| 15 | (((FT=health costs AND AU="home treatment") AND FT=old age psychiatry ) OR FT=homebound ) AND FT=psychogeriatric team | 0 |  |

Formularende
