# Supplementary material for: Effectiveness of multidisciplinary psychiatric home treatment for elderly patients with mental illness: a systematic review of empirical studies
Source: BMC Psychiatry. 2019 Dec 3;19:382. doi: 10.1186/s12888-019-2369-z (PMC6889722; doi:10.1186/s12888-019-2369-z)
Supplement: Supplementary file 2 — Additional file 2. List of search terms. [file 12888_2019_2369_MOESM2_ESM.doc]

**Additional file 2: List of search terms**

**age**

elderly

elderly people

homebound

old age

older adults

**outcome**

effectiveness

effect

costs

economic evaluation

evaluation study

health care costs

quality

quality of care

**type of study**

randomised controlled trial

evaluation

evidence based

meta-analysis

explorative study

quantitative methods

qualitative methods

**type of publication**

original study

research article

review

**alternative search terms**

health care needs

social needs

**mental health**

geriatric mental health

geriatric psychiatry

geronto-psychiatric

mental disease

mental disorder

mental health

mental illness

old age psychiatry

psychiatric illness

psychogeriatrics

social psychiatry

**type of treatment/setting**

assertive community treatment

care management

case management

community-based

community mental health team

community outreach methods

community outreach program

continuing care

health care

home based support

home treatment

home visits

home visiting program

home care

individualised service

intensive psychiatric community care

intervention

mobile

multidisciplinary team

outpatient service

outpatient treatment

patient-centred

psychogeriatric team

psychosocial care/support/approach

treatment

outreach model

intensive community treatment

domiciliary visits
