# Supplementary material for: Effectiveness of multidisciplinary psychiatric home treatment for elderly patients with mental illness: a systematic review of empirical studies
Source: BMC Psychiatry. 2019 Dec 3;19:382. doi: 10.1186/s12888-019-2369-z (PMC6889722; doi:10.1186/s12888-019-2369-z)
Supplement: Supplementary file 3 — Additional file 3. Data extraction form. [file 12888_2019_2369_MOESM3_ESM.doc]

**Extraction sheet**

**Introduction**

- **Unique Identifier**
- **Author(s)**
- **Title**
- **Journal**
- **Country**
- **Year**
- **Study setting (what/where, country, supporter of the team, home treatment, target group)**
- **Type of study**
- **Aim of study**

**Methods**

- **Design (RCT, Longitudinal, Prospective, Retrospective, Case study)**
- **Type of randomisation**
- **Time of examination (baseline-follow-up; Year)**
- **Recruitment to study**

**- Inclusion criteria**

**- Exclusion criteria**

- **Outcome (measures) (what/how)**

**(Length of stay in the hospital, costs, crisis contacts, psychiatric symptoms, functionality…)**

- **Sample (EG/CG/drop outs)**
- **Statistically methods**

**Participants**

- **Age (mean)**
- **Gender**
- **Education level**
- **Religion**
- **Relationship status**
- **Income**
- **Living arrangements**
- **Medication**
- **Multimorbidity**
- **Patient history**
- **Ethnic**
- **Diagnosis**

**Intervention**

- **Model (Home Treatment)**
- **Type of Intervention (psychological, therapy, training…)**
- **Standardised Program**
- **Case load/shared case load**
- **Carer involvement (family members)**
- **Duration**
- **Intensity**
- **Setting (making contact with clients/how, home Treatment)**
- **Team (Profession)**
- **Availability (day/night/weekend, personal contact, telephone contact)**
- **Description usual service**
- **Times of examinations (baseline-follow up)**
- **Referrals**
- **Referrals vs. Gate Keeper**
- **Costs**

**Results**

**Limitations and strengths**

- **Method (e.g. risk of bias)**
- **Statistics (e.g. analysis)**

**Particular characteristics/other**

**Conclusions related to the study**
